# Trans-generational immune priming in honeybees

**DOI:** 10.1098/rspb.2014.0454

**Published:** 2014-06-22

**Authors:** Javier Hernández López, Wolfgang Schuehly, Karl Crailsheim, Ulrike Riessberger-Gallé

**Affiliations:** Department of Zoology, Karl-Franzens University of Graz, Universitätsplatz 2, 8010 Graz, Austria

**Keywords:** immune priming, *Paenibacillus larvae*, artificial rearing, haemocytes, honeybees

## Abstract

Maternal immune experience acquired during pathogen exposure and passed on to progeny to enhance resistance to infection is called trans-generational immune priming (TgIP). In eusocial insects like honeybees, TgIP would result in a significant improvement of health at individual and colony level. Demonstrated in invertebrates other than honeybees, TgIP has not yet been fully elucidated in terms of intensity and molecular mechanisms underlying this response. Here, we immune-stimulated honeybee queens with *Paenibacillus larvae* (*Pl*), a spore-forming bacterium causing American Foulbrood, the most deadly bee brood disease worldwide. Subsequently, offspring of stimulated queens were exposed to spores of *Pl* and mortality rates were measured to evaluate maternal transfer of immunity. Our data substantiate the existence of TgIP effects in honeybees by direct evaluation of offspring resistance to bacterial infection. A further aspect of this study was to investigate a potential correlation between immune priming responses and prohaemocytes–haemocyte differentiation processes in larvae. The results point out that a priming effect triggers differentiation of prohaemocytes to haemocytes. However, the mechanisms underlying TgIP responses are still elusive and require future investigation.

## 1. Introduction

Recent studies involving different species of insects have expanded the knowledge of immunology in invertebrates, but also increased the understanding of its limits [1–7]. Invertebrates were previously believed to rely purely on their innate defences to combat infections [8], and specificity or memory of immune responses were previously considered the hallmark of a highly evolved immune system, only present in vertebrates. Research, however, has revealed that the immune system of invertebrates shares several homologies with that of vertebrates [9–12]. Although the invertebrate immune system lacks lymphocytes and functional immunoglobulins, an increasing number of studies cite induced immune responses in invertebrates which in some cases indicate specificity [13,14]. Other studies have identified haemocytes to be responsible for the specificity of these responses. Cumulatively, these studies show that encounters with a pathogen can enhance the phagocytic activity of haemocytes and mediate specificity in immune protection [15–18]. Moreover, maternal immune experience has been demonstrated to be transmitted to progeny and may therefore have a positive impact on offspring resistance and survival of infections. This phenomenon of trans-generational immune priming (TgIP) has been reported in both vertebrates [19,20] and invertebrates [21–27]. Nevertheless, the magnitude of this response and the underlying molecular mechanisms are still poorly understood in invertebrates.

Honeybees are social insects, forming colonies composed of up to 50 000 individuals or more, residing in a minimal space that offers ideal conditions for the transmission of pathogens and parasites [28]. They show high levels of sociability and physical contact between individuals and environmental homeostasis inside the colony is highly controlled. Offspring are likely to face the same pathogen pressures as queens and could benefit from TgIP effects. In such an environment, the mother queen, upon immunological encounter with a pathogen, could influence the immunity of direct progeny, thus increasing resistance to current infection in the colony.

Here, we investigated the occurrence of TgIP in honeybees by immune-challenging queens with heat-killed bacteria of *Paenibacillus larvae* (*Pl*), causative agent of American foulbrood (AFB) [29] and exposing their first instar larvae to *Pl* spores*.* AFB is considered to be the most threatening bacterial disease of honeybee brood. The spores represent *Pl*'s infectious stage and adult honeybees, which are resistant to infection, serve as vectors within and between colonies, delivering spores to the brood while nursing [30]. During the summers of 2011 and 2012, we conducted a series of experiments to address the existence of TgIP, evaluated the magnitude of such a response and quantified changes in haemocyte populations in honeybee larvae. To the best of our knowledge, for the first time a TgIP effect is demonstrated in honeybees by directly assessing the mortality rate of artificially infected offspring of immune-challenged queens.

## 2. Material and methods

### (a) Insects, bacteria and spore suspensions

All honeybees used in our study belong to *Apis mellifera* subsp*. carnica* and were kept in the garden apiary of the Karl-Franzens University of Graz under normal living conditions. For initial dose-finding experiments that were carried out to determine the required spore dose for infection, offspring from queens of regular colonies (named full colonies) were used. For trans-generational experiments, we used colonies kept in small polystyrene chambers composed of *ca* 1000–2000 individuals each headed by one young naturally multi-mated queen (nucleus colonies). During this study, our goal was to work with colonies as unrelated as possible in order to assure genetic variability of queens. For trans-generational experiments, queens were randomly assigned to one of the following two treatments: challenge with ringer (injected with ringer solution) or challenge with heat-killed *Pl* bacteria (injected with heat-killed *Pl* bacteria). The experimental set-up of the experiments carried out in 2011 and 2012 is summarized in table 1. In summer 2011, *Pl* genotype Eric II (strain CCUG 48972) was used in our experiments and in summer 2012, *Pl* genotype Eric II (strain 233/00) was used [31,32]. Owing to the small size of the colonies used for the priming experiments, which makes a successful overwintering impossible, all colonies were discarded at the end of summer.

**Table 1.** Summary table, including experimental design, colonies, cumulative mortality at day 12, number of larvae and replicates. (Numbers in parenthesis refer to larvae used for each experiment and replicates.)

cumulative mortality at day 12 (number of larvae and replicates per colony)					
				post-challenge (%)	
			pre-challenge (%)	ringer	heat-killed Pl
2011	nucleus colonies	11–1	72.9 (48)	65.1 (48/48/48/48)	—
		11–2	66.7 (48)	—	38.2 (48/48/48)
		11–3	68.7 (48)	—	40.3 (48/48/48)
	full colonies	Hive-20sp	64.6 (48/48)	—	—
2012	nucleus colonies	12–1	54.2 (48/48)	50.7 (48/48/48)	—
		12–2	51 (48/48)	47.9 (48/48/48)	—
		12–3	45.8 (48/48)	—	27.3 (47/43/20)
		12–4	52.1 (48/48)	—	29.9 (48/48/48)
		12–5	45.8 (48/48)	—	19.4 (48/48/48)
		12–6	44.8 (48/48)	—	17.5 (48/48/47)
	full colonies	Hive-20sp	56.2 (48/48)	—	—

Typing of different strains of *Pl* led to the description of four different genotypes (Eric I, II, III and IV), of which Eric I and Eric II are associated with classical AFB outbreaks [33]. Eric I and Eric II show genotype-specific differences in disease progression and in pathogenesis [34]. We chose to work with two strains of Eric II (these strains share the same mechanism of pathogenesis) owing to the high virulence of Eric II at the individual level [29]. As known from sporulating bacteria, *Pl* strains used repeatedly under laboratory conditions may lose the ability to sporulate, which made it necessary for us to work with a different *Pl* strain for the experiments in 2012. The two different strains of Eric II used in 2011 and 2012, respectively, differ solely in the degree of virulence at the individual level (see §3*b*).

To obtain spores, a few colony forming units (CFU) of *Pl* were used to inoculate Columbia sheep blood agar slants and incubated at 34.5°C for 12–14 days. Subsequently, the liquid supernatant was collected, heated for 10 min at 85°C to eliminate vegetative forms (three repetitions with each 10 min at room temperature between treatments) and used to determine spore concentration by cultivating serial dilutions on MYPDG-agar plates. The spore suspension was stored at 4°C and used throughout all experiments.

Previous to any study of the effects of queen priming on larvae, the virulence level of both strains of *Pl* Eric II was assessed by using larvae from four different colonies of regular dimensions. For artificial rearing of honeybee larvae, we used a method by Aupinel *et al*. [35], which was further developed in our laboratory [36] (see the electronic supplementary material). We aimed at finding a spore concentration that produces a larval mortality in a range where any positive or negative effect owing to the priming of queens can be evaluated. For this purpose, we exposed first instar honeybee larvae to different spore loads of *Pl* (exposure bioassays) to obtain the mortality rate due to *Pl* infection at day 12 of the experiment as a function of the spore inoculum per larva (see the electronic supplementary material determination of bacterial virulence and electronic supplementary material, figure S1 for spore doses and related mortality). The experiments were ended at day 12 to minimize the risk of laboratory contamination through spore-forming larval masses.

### (b) Exposure bioassay before the challenge of queens

Before any challenge of queens of nucleus colonies, their offspring were exposed at first instar to the previously determined spore dose of *ca* 20 spores per larva to confirm mortality rates obtained in full colonies (see the electronic supplementary material, figure S1). Artificial larval rearing was followed as described in the electronic supplementary material (see determination of bacterial virulence). This procedure was carried out in three and six nucleus colonies in 2011 and in 2012, respectively. The number of dead larvae was recorded every day for all groups during 12 consecutive days. Artificial rearing leads generally to small losses of larvae in the range of *ca* 10%. To put the number of larvae dying from *Pl* infection in relation to control larvae, the mortality in a control group was always co-assessed. For the experiments, a total of 3154 larvae were artificially reared. For the study, detailed numbers of each group and replicates are given in table 1.

To confirm the inoculum of *ca* 20 spores, infectious diet fed to larvae was plated every time on MYPGD-agar and CFU were counted 6 days later. Larval diet with a 50% content of royal jelly has a strong antibacterial activity. Plating diet on agar allows spores to germinate and first CFU appear after 3–6 days. The growth of *Pl* was confirmed: (i) when no bacterial growth was detected before day 3, which indicated that no significant bacterial contamination was present; and (ii) by occasionally checking the identity of *Pl* using PCR.

### (c) Challenge of queens

Queens of the nucleus colonies were randomly assigned to either a ringer- or *Pl*-challenge group (1 ringer- and 2 *Pl*-challenge queens in 2011 and 2 ringer- and 4 *Pl*-challenge queens in 2012). Queens were chilled on ice for around 5–10 min and injected between the fifth and sixth abdominal tergites using a Hamilton micro-litre syringe. Immune-challenged queens received 2 µl of heat-killed (90°C, 10 min) vegetative forms of *Pl* genotype Eric II (strain CCUG 48972 in 2011 and strain 233/00 in 2012, respectively) suspended in sterile ringer solution at a concentration of 10^8^ bacterial cells ml^−1^ and the efficiency of the process was confirmed by plating out samples of the suspension on MYPGD-agar. Control queens received 2 µl of sterile ringer solution. Following injections, queens were immediately taken off ice and returned to their colonies.

### (d) Exposure bioassays after the challenge of queens

After the challenge of queens, colonies were observed daily and, at first, first instar larvae were grafted and artificially reared and exposed to *Pl* spores as described above. Each experiment consisted of offspring from ringer- or *Pl*-challenged queens that were fed with infectious diet in a concentration of *ca* 20 spores per larva and their mortality rate was compared with the mortality of control groups, which were fed with non-infectious diet. Conditions and rearing methods were exactly the same as for larvae for the exposure bioassay before the challenge of queens. The number of dead larvae was recorded every day for all groups during the following 12 days. To confirm the inoculum of *ca* 20 spores, infectious diet was plated every time on MYPGD-agar, CFU were counted 6 days later and the identity of *Pl* was confirmed as described in §2*b*.

### (e) Larval samples for haemocyte counts

Parallel to the exposure bioassays, larvae of queens challenged with ringer or *Pl* of the six colonies in 2012 were artificially reared in the laboratory under regular conditions (no exposure to spores) and were used on day 6 to perform haemocyte counts using a Bürker-Türk haemocytometer. In this study, we included counts for total haemocytes and for differential haemocytes. Here, we refer to differential haemocytes to all those other haemocyte types observed which are no longer in the prohaemocyte stadium but have reached a different developmental stadium (granulocyte, oenocytoid or plasmatocyte). Total haemocyte counts include prohaemocytes and all differential haemocytes observed.

Haemolymph samples were extracted by puncturing larvae with a sterile needle and 1 µl of haemolymph was collected by using sterile glass micro capillaries. A Bürker-Türk haemocytometer was then used to determine total haemocyte counts and differential haemocyte counts in a 1/10 dilution factor. Cell counts were carried out 5–10 min after filling the haemocytometer. Differential haemocytes were identified by their morphology as described in the literature [37,38].

### (f) Statistical analysis

The software package SPSS v. 19 was used for statistical analyses. A Cox regression analysis was performed to estimate differences in larval mortality among colonies considering the effect of the three different parameters: (i) type of treatment, (ii) strain, and (iii) queens used. Regarding levels of haemocytes, an examination of the histograms of the distribution of total haemocyte counts and differential haemocyte counts showed a deviation from normal distribution. Therefore, non-parametric methods (Mann–Whitney *U*-test, Kruskal Wallis one-way ANOVA) were used for statistical analysis.

## 3. Results

### (a) Determination of bacterial virulence

Mortality rates assessed in initial experiments in full colonies of larvae exposed to different doses of *Pl* spores for both strains are plotted in the electronic supplementary material, figure S1. The results allowed us to select an adequate spore dose of *ca* 20 spores per larva for subsequent experiments, which causes a mortality rate of around 65% for *Pl* strain CCUC 48972 and a mortality of 50% for *Pl* strain 233/00, respectively. In subsequent experiments, nucleus colonies composed of 1000–2000 individuals and a young multi-mated queen were exclusively used. This points to a different virulence of the test strains as shown by their different absolute mortality rates.

### (b) Exposure bioassay before the challenge of queens

**Figure 1.**
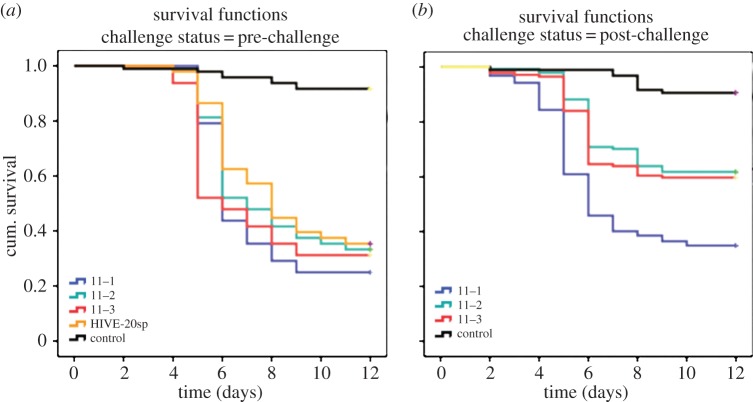
Survival profile of larvae of nucleus colonies exposed to 20 spores of *Pl* Eric II-strain CCUG 48972 in 2011. (*a*) Pre-challenge: colony 11–1; 11–2 and 11–3 (*n* = 48 per colony, one time tested); control pre-challenge (*n* = 48 + 48, larvae from all three nucleus colonies fed with non-infectious diet, two replicates throughout this part of the experiments). Hive-20sp: full colony from preliminary screening of bacterial virulence (*n* = 48 + 48, two replicates). (*b*) Post-challenge: colony 11–1 ringer-treated queen (*n* = 48 + 48 + 48 + 48, four replicates); 11–2 *Pl*-treated queen (*n* = 48 + 48 + 48, three replicates); 11–3 *Pl*-treated queen (*n* = 48 + 48 + 48, three replicates); control post-challenge (*n* = 144, larvae of all three nucleus colonies fed with non-infectious diet, four replicates throughout this part of the experiments). (Online version in colour.)

**Figure 2.**
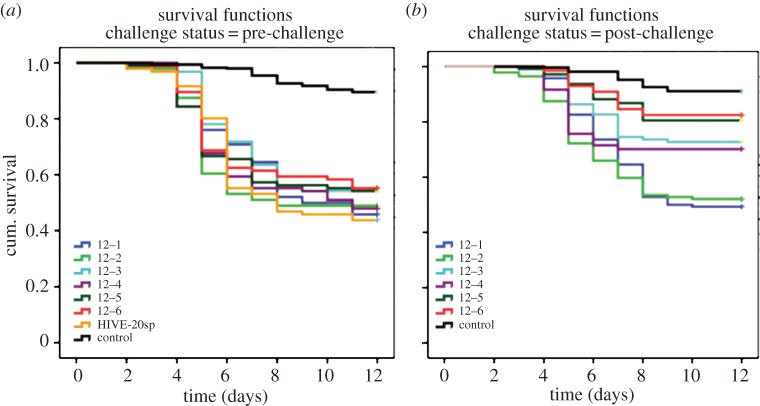
Survival profile of larvae of nucleus colonies exposed to 20 spores of *Pl* Eric II-strain 233/00 in 2012. (*a*) Pre-challenge: colony 12–1; 12–2; 12–3; 12–4; 12–5; 12–6 (*n* = 48 + 48 per colony, two replicates); control pre-challenge (*n* = 355, larvae of all six nucleus colonies fed with non-infectious diet, seven replicates throughout this part of the experiments). Hive-20sp: full colony from preliminary screening of bacterial virulence (*n* = 48 + 48, two replicates). (*b*) Post-challenge: colony 12–1 ringer-treated queen (*n* = 48 + 48 + 48, three replicates); 12–2 ringer-treated queen (*n* = 48 + 48 + 48, three replicates); 12–3 *Pl*-treated queen (*n* = 47 + 43 + 20, three replicates); 12–4 *Pl*-treated queen (*n* = 48 + 48 + 48, three replicates); 12–5 *Pl*-treated queen (*n* = 48 + 48 + 48, three replicates); 12–6 *Pl*-treated queen (*n* = 48 + 48 + 47, three replicates); control post-challenge (*n* = 338, larvae of all six colonies fed with non-infectious diet, eight replicates throughout this part of the experiments). (Online version in colour.)

A Cox regression analysis revealed no statistically significant differences in mortality between larvae of full colonies (named Hive-20sp) and nucleus colonies for 2011 and 2012, respectively (table 2). The data also showed that there is no significant difference in the mortality rate of larvae from different queens for both the 2011 and 2012 experiments (table 2). For survival profiles of larvae of nucleus colonies and details regarding replicates and number of larvae used, see figures 1*a* and 2*a* for colonies in 2011 and 2012, respectively. The mortality rate obtained at a dose of *ca* 20 spores (Hive-20sp) was later confirmed in three independent colonies in 2011 (one time tested, three colonies) and six independent colonies in 2012 (two times tested, six colonies). A Cox regression analysis revealed no significant differences in mortality rates of larvae and also no effect of queens on the mortality rate. Hence the mortality rate did not depend on the fact that queens were recruited from different colonies. This strongly confirms that *Pl* strain CCUC 48972 is responsible for a mortality of around 65% (2011) and that *Pl* strain 233/00 is responsible for a mortality of 50% (2012) when applied at a dose of *ca* 20 spores per larva independently of the colony.

**Table 2.** Results of the Cox regression analysis for mortality of larvae of full colonies (Hive-20sp) and nucleus colonies exposed to a dose of 20 spores per larva before the treatment of queens.

	Wald's *χ*²	d.f.	*p*-value	relative risk	95% CI for relative risk	
					lower	upper
treatment 20 spores (2011)	0.163	1	0.687	0.761	0.201	2.873
queens (2011)	0.178	3	0.981			
treatment 20 spores (2012)	1.328	1	0.249	0.463	0.125	1.714
queens (2012)	4.974	7	0.663

### (c) Challenge of queens

The procedure of the challenge of queens is generally well tolerated. From 3 years of experiments, we found that the caging of challenged queens before returning them to the nucleus colonies leads to 100% acceptance of queens, as found during experiments in 2013. However, in 2011 and 2012, we were not completely aware of this fact and some queens were rejected after returning them into the nucleus colonies.

### (d) Exposure bioassay after the challenge of queens

A Cox regression analysis showed significant differences in the mortality rate of larvae regarding treatment of their mothers (table 3), indicating that a challenge of queens with heat-killed *Pl* significantly reduces mortality rates upon an exposure to *Pl* spores. This is not dependent on the origin of queens (table 3). As expected, the strain type has an influence on mortality rates obtained. As previously mentioned, the strain type used in 2011 causes a slightly higher mortality in the exposure bioassays than the strain type used in 2012 (table 3). For survival profiles per colony, details regarding replicates, and number of larvae used, see figures 1*b* and 2*b* for colonies in 2011 and 2012, respectively.

**Table 3.** Results of the Cox regression analysis for mortality of larvae of nucleus colonies exposed to a dose of 20 spores per larva after treatment of queens with ringer or bacteria.

	variables in the equation^b^			
	Wald's *χ*²	d.f.	*p*-value	exp (B)
treatment ringer_*Pl*	26.994	1	0.000	0.297
queens	10.131	6^a^	0.119	
strain	16.056	1	0.000	0.383

^a^Degree of freedom reduced because of constant or linearly dependent covariates.

^b^Constant or linearly dependent covariates queen(3) = 2 – year – queen(1) – queen(2); ringer_*PI* = 2 – queen(1) – queen(4) – queen(5).

A Cox regression analysis showed no effect of ringer-injection on mortality of larvae compared with mortality rates obtained for the same colonies before injection of ringer. As expected, the origin of the queens has no effect on the results and the two strain types used show a variation in virulence (table 4). This result allows us to confirm that the ringer-challenge has no effect on mortality rates obtained before and after the injection of queens, and, strongly reinforces that the reduction of mortality observed for experimental colonies is owing to the injection of heat-killed bacteria. Hence, we had a total of three ringer control colonies and six experimental colonies in 2 years where the effect of TgIP is consistent and the strain type used only affects the cumulative mortality at the end of day 12. Cumulative mortalities for all colonies used in the experiments are shown in table 1.

**Table 4.** Results of the Cox regression analysis for mortality of larvae of nucleus colonies exposed to a dose of 20 spores per larva after treatment of queens with ringer.

	variables in the equation^b^					
	Wald's *χ*²	d.f.	*p*-value	relative risk	95% CI for relative risk	
					lower	upper
ringer_treatment	0.800	1	0.371	0.909	0.737	1.121
queens	0.065	1^a^	0.799			
strain	11.775	1	0.001	0.656	0.515	0.835

^a^Degree of freedom reduced because of constant or linearly dependent covariates.

^b^Constant or linearly dependent covariates queen(1) = 2 – year.

**Figure 3.**
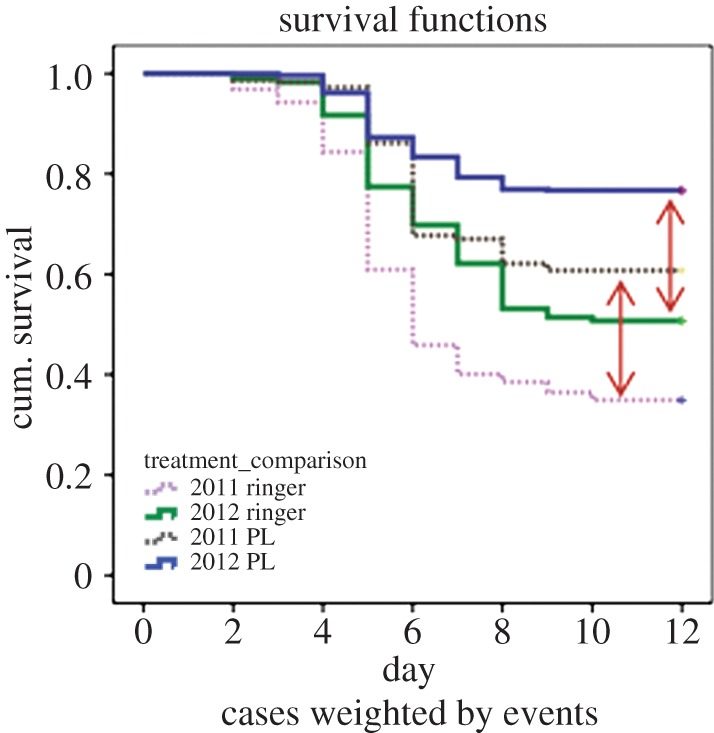
Survival profile of all larvae of nucleus colonies after challenge of queens with ringer or *Pl* bacteria. Arrows indicate the difference in mortality for colonies treated with ringer versus *Pl*. (Online version in colour.)

Survival functions for all larvae of each of the complete number of offspring from ringer-challenged or *Pl*-challenged queens in 2011 and 2012 are presented in figure 3. Cumulative larval mortality for ringer-challenged queens is 65.1% and for *Pl*-challenged queens 39.2% in 2011, which indicates a reduction in larval mortality of 25.9%, whereas in 2012 cumulative larval mortality for ringer-challenged queens is 49.3% and for *Pl*-challenged queens 23.2%, which indicates a reduction in larval mortality of 26.0%. The effect of *Pl*-challenge is consistent independently of the strain type, which causes a deviation in mortality owing to the higher virulence of the strain used in 2011.

To equalize the paternal influence on the genetic constitution of larvae, we used multi-mated queens to minimize the individual male genetic donation. Interestingly, we noted that upon exposure to *Pl* spores, segregation among the four colonies in 2012 headed by *Pl*-challenged queens into two groups with different larval mortality rates occurred (12–3 and 12–4 versus 12–5 and 12–6, figure 2*b*). Since experimental procedures were strictly the same for all colonies in both seasons, we can hypothesize that either differences among the effector genes of immune responses prevail or that the individual history of pathogen exposure of the parents contributes to this variation. Further, environmental conditions within each nest could also have an influence on the observed segregation.

### (e) Haemocyte levels in larvae

First, differences between colonies within the two treatment groups (two colonies with ringer-challenged queens and four colonies with *Pl*-challenged queens) were analysed. Results showed no statistically significant differences for total haemocyte counts (ringer colonies: Mann–Whitney *U*-test, *p* = 0.902; *Pl* colonies Kruskal Wallis one-way ANOVA *p* = 0.982) and differential haemocyte counts (ringer colonies: Mann–Whitney *U*-test, *p* = 0.456; *Pl* colonies Kruskal–Wallis one-way ANOVA *p* = 0.135).

Because no significant differences were observed, we combined the two ringer-treated colonies and the four *Pl*-treated colonies, respectively, and compared differences in total (prohaemocytes, granulocytes, oenocytoids and plasmatocytes) and differential (granulocytes, oenocytoids and plasmatocytes) haemocyte counts for these two groups of colonies. No statistically significant differences were found for total haemocyte counts (Mann–Whitney *U*-test *p* = 0.735). An average of 3826.8 (s.d. = 1840.5) total haemocyte counts per µl of haemolymph was found in larvae of the ringer-challenged queens and 3675.6 (s.d. = 2082.1) in the *Pl*-challenged queens, respectively.

Interestingly, we found statistically significant differences between the differential haemocyte counts for the two treatment groups (Mann–Whitney *U*-test *p* = 0.003). The levels of differential haemocytes were higher in larvae of *Pl*-challenged queens as compared with those in larvae of ringer-challenged queens. Differential haemocytes, which include all other haemocytes types except for prohaemocytes, were clearly identified as oenocytoids, plasmatocytes and granulocytes in all types of larvae. The percentage of differential haemocytes of total counts was 0.98% in larvae of ringer-challenged queens compared with 3.22% in larvae of *Pl*-challenged queens. This translates to a threefold increase in the number of differential haemocytes in the haemolymph (electronic supplementary material table S2). These results support a previously described role of haemocytes in TgIP in invertebrates [27,39].

## 4. Discussion

The aim of our study was to seek evidence of TgIP in a scenario as close to natural conditions as possible by directly evaluating offspring resistance to a specific bacterial infection and by quantifying the cellular response. This aim complements approaches pursued in other works, where immune parameters such as levels of antimicrobial peptides in offspring were measured [22,23,40]. The latter studies demonstrated that induced levels of antimicrobial peptides in offspring are higher when their parents received an immune challenge. In line with this, recent work on *Tenebrio molitor* focused on determining associated costs due to the induced immune response produced in progenitors. Apparently, maintaining enhanced levels of immune defence showed interdependence with other fitness-related traits like a longer time of development in primed offspring [23,27] or trade-offs between maternal immunity, egg production and protection [41,42].

So far, the evaluation of the individual benefit of TgIP responses, i.e. how strong a TgIP effect confers protection to offspring in terms of reduced mortality, has not been a primary goal of investigation. We aimed at filling this gap of knowledge through quantification of maternal transfer in terms of offspring's survival rate upon pathogen exposure. Our observations reveal an immune priming-dependent maternal effect on offspring immunity that strongly increases their survival rate by *ca* 26.0% to AFB infection. Many questions have not been fully answered yet, e.g. whether the priming effect observed in offspring is a consequence of a general immune activating in the queen (e.g. through antimicrobial peptides) or belongs to a more complex and targeted pathogen-dependent response (e.g. through haemocytes). Besides, nothing is known about the transfer mechanism of a TgIP response from mother to offspring in invertebrates.

Evidently, there is a general interest in investigating fitness costs related to TgIP, which is of great importance to better understand the interdependence and coevolution of a host–parasite system in insects. Besides, the physiological feasibility of immune investments has to be taken into account. An investment in immune competence could be beneficial as long as the infection is present in the colony and as long as it does not exert excessive negative effects on the queen's fitness. In the case of severe colony infection, a high investment in immunity might be unavoidable to prevent the loss of the whole colony. Thus, queens may possess mechanisms to somehow estimate the magnitude of a current infection and act accordingly [41].

Honeybees possess only one-third of genes involved in immunity in their genome as compared with *Anopheles* or *Drosophila*. Nonetheless, the four known pathways implicated in immunity in invertebrates remain functional in honeybees [6]. Compensation for this lack of genes has been attributed to the existence of social immunity [43]. Considering fitness costs, this would suggest that a short-lived worker honeybee is not expected to display a complex immune response as that found in other insects. If, however, long-lived honeybee queens maintained even part of these immunological features, the queen as a single individual could positively influence the immunological status of the whole colony. Hence, this finding could also have a major impact on the beekeeping industry to combat diseases challenging this indispensable pollinator by naturally conferring resistance to infections (e.g. the development of immunization programmes in apiculture).

While the mechanisms underlying this transmission of immunity are not fully understood in invertebrates, results in bumblebees point out the existence of a factor transmitted via the eggs [40]. Therefore, in future, an in-depth study of the genetic expression and epigenetic factors that might be involved in this transfer of immunity from primed progenitors to offspring may reveal molecular characteristics of such process.

Parallel to the exposure bioassays demonstrating TgIP, we examined the degree of differentiation of haemocytes in larval immunity. Haemocytes are the immune cells of the innate immune system of invertebrates and prohaemocytes are the non-immune competent precursor cells of all haemocyte populations, which we name here differential haemocytes. Our results point out that maternal immune experience triggers differentiation of prohaemocytes to prepare offspring for a prevailing pathogen. Thus, larvae containing enhanced levels of immune competent cells could possess the ability to react more rapidly to infection, which results in reduced mortality rates. Even though in larvae a direct contact haemocyte-*Pl* bacterium is unlikely, immune competent haemocytes associated with the gut tissue actively participate in the production of antimicrobial peptides and secretion towards epithelial cells of the gut tissue and gut lumen, where the infection starts [44].

On a related topic, a transfer of immunity to offspring upon viral infections has been shown for Lepidoptera [24]. Since high virus titres of different viruses have been found co-infecting colonies suffering from *Varroa* and colony collapse disorder [28], experimental verification of such a transfer of immunity upon viral infection in honeybees would be of great interest.
